# Acute Kidney Injury

**DOI:** 10.1155/2015/595947

**Published:** 2015-01-06

**Authors:** Raúl Lombardi, Emmanuel A. Burdmann, Alejandro Ferreiro, Fernando Liaño

**Affiliations:** ^1^Department of Critical Care Medicine, Servicio Médico Integral, Montevideo, Uruguay; ^2^Division of Nephrology, University of Sao Paulo Medical School, Sao Paulo, Brazil; ^3^Department of Nephrology, Universidad de la República and Department of Nephrology, CASMU, Montevideo, Uruguay; ^4^Department of Nephrology, Hospital Universitario Ramón y Cajal, Instituto Ramón y Cajal de Investigación Sanitaria (IRYCIS), RedinRen, CIFRA, 28034 Madrid, Spain

Acute kidney injury (AKI) is a frequent and serious clinical condition which is associated with poor outcomes, including high mortality rate. Classically, it was considered as an acute condition, potentially reversible with full restitution if patient survives the acute phase of the disease. However, recent epidemiologic and observational studies underscore the association of an episode of AKI with long-term adverse outcomes such as chronic kidney disease, end-stage renal disease, cardiovascular events, and premature death.

The increasing incidence of AKI, the association with severe in-hospital complications and long-term outcomes and death, the rise in costs, and the potentially preventable nature of AKI make it as a major public health issue, raising the interest of investigators in the field. Focusing on avoidance of the syndrome or its complications, the burden of the disease that is estimated in more than 13 million people/year all around the world could be effectively reduced.

In this special issue devoted to AKI, the potential protective effects of diverse interventions were explored in five basic research studies. Y.-Y. Chen et al. explored the effect of 2-methoxyestradiol against ischemia/reperfusion injury. The protective effect of the angiotensin-receptor blocker olmesartan in rats exposed to tacrolimus is addressed by N. O. Al-Harbi et al. The influence of acute superoxide radical scavenging on systemic hemodynamic and kidney function was investigated by Z. Miloradović et al. in a model of induced postischemic AKI. Z. O. Ibraheem et al. evaluated the impact of high fructose feeding in rat model of gentamicin induced nephrotoxicity. Finally, W. Zhang et al. addressed the feasibility and efficacy of hypoxia preconditioning to enhance MSC-based therapy of AKI.

Contrast induced nephropathy (CIN) was addressed in three clinical studies. The beneficial effect of atorvastatin and rosuvastatin administered at high doses and before iodinated contrast administration was addressed by M. Peruzzi et al. concluding that they have a consistent and beneficial preventive effect on CIN and may actually halve its incidence. The incidence of CI-AKI and ERD in patients who received iodixanol (isoosmolar) versus iohexol (low-osmolar) during angiography for cardiac indication was examined by H.-R. Chua et al. who found advanced age, emergent cardiac conditions, and critical illness as stronger predictors of CI-AKI. J. Malyszko et al. sought cytokine midkine as a potential early marker of renal injury after contrast administration.

In a series of critically ill patients with AKI, C.-M. Zheng et al. found that anion gap value predicts short-term mortality in patients with metabolic acidosis and AKI, whereas the strong ion gap value predicts both short-term and long-term mortality among such patients. Dr. H. E. Liu et al. explored genetic polymorphisms of ERCC1 and TP53 as risk factors for cisplatin- and carboplatin-induced nephrotoxicity. Dr. B. B. Albino et al. wondered whether the duration of extended dialysis was associated with complications in AKI patients.

The frequency of TGF-*β* and IFN-*γ* genotype as risk factors for AKI and death in ICU patients is explored by C. C. Grabulosa et al. who found no association with these outcomes. S. N. Fernández et al. compared the efficacy of heparin and citrate for anticoagulation in critically ill children on CRRT and found that citrate is a safe and effective anticoagulation method. In a large cohort of AKI patients V.-C. Wu et al. found a decreasing risk of long-term ESRD and mortality in patients surviving an episode of AKI-requiring dialysis, AKI nonrequiring dialysis, and no AKI, respectively.

Finally, pathophysiology of cisplatin-induced AKI and nephrotoxicity of contrast media were reviewed by A. Ozkok and C. L. Edelstein and M. Andreucci et al., respectively.

We hope this special issue on AKI collaborates in raising the awareness on this clinical condition that challenges not only nephrologists and intensivists who mostly deal with patients with AKI but also primary care physicians, clinicians, surgeons, radiologists, and those who are “in the right place at the right time” to prevent the onset of AKI or, even more, the disease leading to AKI.

*Raúl Lombardi* *Emmanuel A. Burdmann* *Alejandro Ferreiro* *Fernando Liaño*

